# Exploiting p53 Status to Enhance Effectiveness of Chemotherapy by Lowering Associated Toxicity

**DOI:** 10.18632/oncotarget.247

**Published:** 2011-03-28

**Authors:** Linda S. Steelman, Alberto M. Martelli, Ferdinando Nicoletti, James A. McCubrey

**Affiliations:** ^1^ Department of Microbiology & Immunology Brody School of Medicine at East Carolina University Greenville, NC 27858 USA; ^2^ Dipartimento di Scienze Anatomiche Umane e Fisiopatologia dell'Apparato Locomotore Università di Bologna, Bologna, Italy; ^3^ IGM-CNR, Sezione di Bologna, C/o IOR, Bologna, Italy; ^4^ Department of Biomedical Sciences University of Catania, Catania, Italy

The central dilemma in chemotherapy is to eliminate the rapidly proliferating cancer cells, while leaving the “normal” cells alone as much as possible. Limiting the detrimental side effects of mitotic inhibitors on normal cells could significantly enhance successful chemotherapy [1]. Mitotic inhibitors often kill normal proliferating (cycling) cells such as bone marrow cells, hair follicles, mucosal and epithelial cells [1-3]. These side effects can be severe in some cases and limit the benefits of chemotherapy as well as suppress the desirability of this type of cancer therapy by certain patients. A central question remains how we can improve on chemotherapy but reduce its detrimental side effects.

Approximately 50% of human cancers have mutations which eliminate the functions of p53. However, the normal cells in these cancer patients possess wild-type (WT) p53. p53 is often activated by chemotherapeutic drugs [1,4,5]. The exciting studies by Apontes et al, published in Oncotarget [1], convincingly demonstrate that treatment of cells containing WT p53 but not cells containing mutant p53 with the MDM-2 inhibitor Nutlin-3a [6] would protect p53 WT cells put not p53 mutant cells from the effects of mitotic inhibitors such as paclitaxel and nocodazole. p53 is normally not a stable protein due to its ubiquitination by the MDM2 ubiquitin ligase. Nutlin-3a acts by inhibiting MDM2 which results in p53 stabilization. In essence, these important results stem from the basic biological fact that mitotic inhibitors cannot cause mitotic arrest in cells that do not enter mitosis. Thus in normal cells, a transient arrest in either G_1_ or G_2_ stimulated by Nutlin-3a treatment, will protect them from the catastrophic mitotic arrest induced by mitotic inhibitors such as paclitaxel or nocodazole.

**Figure 1:**
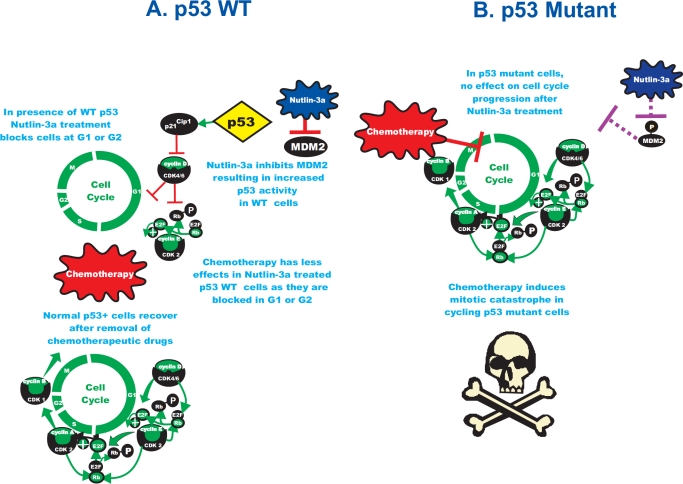
Exploiting the p53 Gene Status to Induce Mitotic Catastrophe in p53 Mutant Cells but not in Normal Cells On the left hand side of the figure labeled A, cells with WT p53 undergo cell cycle arrest upon treatment with Nutlin-3a. However if they are then treated with chemotherapeutic drugs they do not under go mitotic catastrophe. If the chemotherapeutic drug is removed effectively, the normal cells will recover, resume cell cycle progression and grow. On the right hand side of the figure labeled B, in p53 mutant cells, the cells do not undergo cell cycle arrest upon treatment with Nutlin-3a. Then upon treatment with chemotherapeutic drugs, the cycling cells undergo mitotic catastrophe.

Importantly the clinically-approved and readily-used mTOR inhibitor rapamycin potentiated the protective effects of Nutlin-3a in cells with WT p53. Moreover, the anti-diabetic drug metformin would interact with rapamycin in low glucose cell culture conditions to protect cells with WT-p53 but not cells with mutant p53. Metformin also inhibits the effects of mTOR and reduces glucose and insulin levels [7, 8]. Previous studies have indicated that many cancers are mTOR dependent [9]. Indeed, mTOR hyperactivation may contribute to cancer growth, obesity, diabetes as well as premature aging. Many cancers are sensitive to mTOR inhibitors [9-11]. In addition, these same mTOR inhibitors may suppress premature aging and some are employed to treat diabetes and prevent/suppress obesity (metformin). Why are these observations presented by Apontes *et al* so important? These results indicate that it may be possible to increase the effectiveness of chemotherapy by pre-treatment of cancer patients with drugs such as Nutlin-3a or combinations of rapamycin and metformin to preserve the potential of the normal cells to recover after chemotherapeutic drug treatment. That is the normal cells will undergo G1 or G2 arrest and not be sensitive to the effects of chemotherapeutic drugs (See Figure 1). In contrast, cells with mutant p53 will not undergo G1 or G2 arrest and they will be sensitive to the effects of the chemotherapeutic drugs. The authors have proposed potential clinical trials to test these important hypotheses.

**Figure 2:**
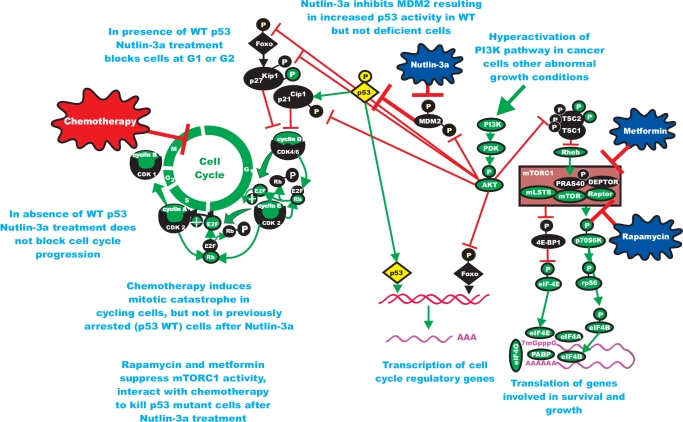
Enhancing Effects of Chemotherapy on Induction of Mitotic Catastrophe in p53 Mutant Cells by Drugs Targeting mTORC1 and MDM2 and Suppressing Detrimental Effects of Chemotherapy in p53 WT Cells Metformin and Rapamycin both suppress mTORC1 but by inhibiting different molecular targets. In the studies by Apontes et al, they have shown that both of these mTORC1 inhibitors can interact with Nutlin-3a to protect p53 WT cells but kill p53 mutant cells. Cancer cells are highly sensitive to drugs which target mTORC1, due to the Warburg effect.

There are two critical keys to these important observations, cancer cells have two potential Achilles heels, they are often mutant at p53 and they also require high concentrations of glucose to proliferate (Warburg effect) via activation of glycolysis followed by lactic acid fermentation. Apontes *et al* exploited the p53 mutation effect by determining the ability of the MDM-2 inhibitor Nutlin-3A to protect three different human cell lines with WT p53 cells: WI-38t (fibroblast immortalized with telomerase), retinal pigment epithelial (RPE) ARPE-19, and normal kidney epithelial (NKE) by pretreatment from the cytotoxic effects of paclitaxel and nocodazole. Nutlin-3a causes G1 and/or G2 arrest in cells with WT p53, but not in cells with mutant p53 (MDA-MB-231, metastatic breast cancer cell line with mutant p53). Thus the cancer cells with mutant p53 were sensitive to the effects of paclitaxel and nocodazole and underwent lethal mitotic arrest even after the removal of the mitotic inhibitors and did not regrow when the drugs were removed. In contrast, cells with WT p53 did not undergo lethal mitotic arrest and recovered and formed colonies. Likewise a combination of rapamycin and metformin induced G1 and G2 arrest in normal cells and protected them from the mitotic inhibitors (See Figure 2). These results were also observed in low glucose conditions which are toxic for the cancer cells. Other studies by this same research group have demonstrated the importance of the p53 and mTOR pathways in regulating senescence and quiescence [12].

While DNA damaging drugs will induce p53 in cells with WT p53, which does to an extent provide some protection to the cells, the effects of DNA damaging drugs are not always reversible. Thus more effective and reversible inducers of p53 have been desired. The small molecule inhibitor Nutlin-3a is an effective inducer of p53 in cells with WT p53 which is more reversible [6,12].

The mTOR pathway is critical in nutrient sensing [9-11]. It can be regulated by the drugs rapamycin or metformin which act at different points in the pathway. Rapamycin targets the key complex mTORC1 which is very important in the regulation of translation of mRNAs critical in cell growth and survival. Rapamycin is used to treat organ transplant patients and is also being evaluated as an anti-cancer and anti-aging drug. Rapamycin would further potentiate the protective effects of Nutlin-3a on cells with WT-p53. Furthermore the combination of rapamycin and metformin would further increase the protective effect. This is most likely due to the different molecular effects of rapamycin and metformin on the mTOR complex. Rapamycin blocks mTORC1 activity [10, 11], while metformin blocks mTORC1 activity by perhaps multiple mechanisms depending upon the cell type. In some cases metformin may affect Rag GTPases [13] while in other cases metformin may inhibit the master metabolic regulator, energy-sensing AMP-dependent protein kinase (AMPK) which normally activates TSC-2 and inhibits Raptor [14]. Metformin and rapamycin also protected normal cells in low glucose conditions. In contrast, the malignant cells, due to their dependence on high concentrations of glucose for rapid growth, were very sensitive to the effects of metformin and rapamycin in low glucose conditions suggesting that dietary (caloric) restrictions may aid in certain cancer therapies. Indeed, it has become clear over the past few years that obesity can contribute to cancer [7,8] and lowering caloric intake may augment cancer therapies [15, 16]. High caloric intake increases mTOR activity and can contribute to insulin-resistance, diabetes, obesity and augment cancer growth and premature aging.

The results presented in the exciting manuscript by Apontes et al, provide a medical hypothesis by which inducing p53 by pre-treatment with Nutlin-3a in cancer patients may synergize with two clinically approved drugs which target the mTOR pathway, rapamycin and metformin to save the normal cells but kill the malignant cells upon chemotherapeutic drug treatment.
